# A head mounted device stimulator for optogenetic retinal prosthesis

**DOI:** 10.1088/1741-2552/aadd55

**Published:** 2018-10-09

**Authors:** Ahmed Soltan, John Martin Barrett, Pleun Maaskant, Niall Armstrong, Walid Al-Atabany, Lionel Chaudet, Mark Neil, Evelyne Sernagor, Patrick Degenaar

**Affiliations:** 1 School of Engineering, Newcastle University, Newcastle upon Tyne, NE1 7RU, United Kingdom; 2 Institute of Neuroscience, Newcastle University, Newcastle upon Tyne, NE1 7RU, United Kingdom; 3 Tyndall Institute, University of Cork, Cork, Republic of Ireland; 4 C4 Sightcare Ltd, Northumberland House, Newcastle upon Tyne, NE1 8ER, United Kingdom; 5 Department of Biomedical Engineering, Helwan University, Helwan, Egypt; 6 Blackett Laboratory, Department Physics, Imperial College, London SW7 2AZ, United Kingdom; patrick.degenaar@newcastle.ac.uk

**Keywords:** retinal prosthesis, visual prosthesis, optogenetics, electronics, regulatory, channelrhodopsin, retinitis pigmentosa

## Abstract

*Objective*. Our main objective is to demonstrate that compact high radiance gallium nitride displays can be used with conventional virtual reality optics to stimulate an optogenetic retina. Hence, we aim to introduce a non-invasive approach to restore vision for people with conditions such as retinitis pigmentosa where there is a remaining viable communication link between the retina and the visual cortex. *Approach*. We design and implement the headset using a high-density *µ*LED matrix, Raspberry Pi, microcontroller from NXP and virtual reality lens. Then, a test platform is developed to evaluate the performance of the headset and the optical system. Furthermore, image simplification algorithms are used to simplify the scene to be sent to the retina. Moreover, *in vivo* evaluation of the genetically modified retina response at different light intensity is discussed to prove the reliability of the proposed system*. Main results*. We demonstrate that in keeping with regulatory guidance, the headset displays need to limit their luminance to 90 kcd m^−2^. We demonstrate an optical system with 5.75% efficiency which allows for 0.16 mW mm^−2^ irradiance on the retina within the regulatory guidance, but which is capable of an average peak irradiance of 1.35 mW mm^−2^. As this is lower than the commonly accepted threshold for channelrhodopsin-2, we demonstrate efficacy through an optical model of an eye onto a biological retina. *Significance*. We demonstrate a fully functional 8100-pixel headset system including software/hardware which can operate on a standard consumer battery for periods exceeding a 24 h recharge cycle. The headset is capable of delivering enough light to stimulate the genetically modified retina cells and also keeping the amount of light below the regulation threshold for safety.

## 1. Introduction

According to the World Health Organization (WHO) in 2014, there are 39 million blind people worldwide [1]. The primary conditions [2] are cataracts, glaucoma, diabetic retinopathy and age-related macular degeneration. For each, there are treatments available at varying levels of success. However, for retinitis pigmentosa (prevalence 1:3000) there is currently no treatment. Those afflicted progress from night blindness through tunnel vision and eventually complete visual loss around middle age. Retinal prosthetics [3] therefore holds the potential to restore lost vision. It can primarily work with conditions such as retinitis pigmentosa where there is a remaining viable communication link between the retina and visual cortex.

The basic concept of any visual prosthesis is to acquire the visual scene through an imaging device, process it in a way to best communicate with the human visual system and then stimulate the remaining nervous tissue. It is in this latter part where the primary variance between different groups lies. Epi-retinal devices such as presented by da Cruz *et al* [4] place electrodes between the vitreous humour and the retina. Sub-retinal devices such as presented by Edwards *et al* [5] place electrodes behind the retina. A further approach is to place the electrodes in a sub-choroidal arrangement (e.g. by Shivdasani *et al* [6]). Each of these approaches has inherent advantages and disadvantages, and further reading can found in a recent detailed review by Goetz *et al* [7].

In order to present useful information, the prosthetic device must present a spatial distribution of ‘pixel’ stimuli which provide spatial, temporal and contrast information. Zhao *et al* [8] demonstrated that 144-pixel stimuli would be sufficiently effective at reading individual Chinese. Similarly, Cha *et al* [9, 10] researched reading tasks with Latin characters indicating that 625-pixel stimuli would provide a minimum. Although in both cases, reading text from individual characters would be laborious. Cha *et al* [11] followed up with another paper exploring pixel count for mobility and again found 625-pixel stimuli sufficient for basic mobility. Thompson *et al* and Hu *et al* [12, 13] found that rudimentary facial detection could be achieved with an array of 32  ×  32 (1024) pixel stimuli. Though this was assuming the face would always fill the field of view. In addition to spatial resolution, we have found in our own work [14] that contrast sensitivity is also a very important determinant of visual capability. Hu *et al* [13] also considered contrast and did their experiments with eight distinctive grey levels (3 bits dynamic range), which can be defined as a minimum requirement.

There is steady progress in electrode stimuli form of retinal prosthesis, but achieving higher resolutions and high contrast is proving challenging. An alternate technique for retinal stimulation is to use optogenetics to photosensitise remaining cells and stimulate them with light. Optogenetics uses gene-therapy techniques to incorporate a gene to produce light-sensitive ion channels or pumps, thus rendering the cell activity controllable by light. The technique stems from the discovery of channelrhodopsin-2 (ChR2) in 2003 by Nagel *et al* [15]. Since then, a significant number of variants have been developed as can be seen in a recent review by Bergs *et al* [16]. Klapper *et al* [17] and Barrett *et al* [18] have recently reviewed efforts to date in optogenetically sensitising the retina. In short, as for sub and epi-retinal prosthetics, it is conceivable to stimulate the retinal ganglion cells (RGCs) and the bipolar cells. But in addition, in late stage retinitis pigmentosa, the light sensing cone cells lose their outer segments and thus light sensing capability but are otherwise still viable. Busskamp *et al* [19] therefore demonstrated that it is possible to restore some light sensitivity by incorporating halorhodopsin into degenerate cone cells. Though the caveat is that this would represent only a small tunnel vision and the long-term viability of such cells is currently unknown. One of the key functional advantages of the optogenetic technique is that it is possible to target different sub-circuits with different wavelength-sensitive opsins. A key demonstration of this has been the targeting of ON-type (but not OFF-type) bipolar cells by Cronin *et al* [20]. As information in the retina is differential between ON and OFF cell types, specific targeting could result in much better contrast than ubiquitous stimulation.

The key caveat to the optogenetic technique is that optically sensitized cells require considerable irradiance to be activated by light. The threshold in dissociated culture is typically taken at 0.7 mW mm^−2^ [22]. However, *in vivo*, photoresponse typically follows an s-curve of response with the log of irradiance over 2–3 log units as can be seen in table 1 (note this is converted from photons/cm^2^/s in the original papers). For further note, Barrett *et al* [23] demonstrated photoresponses below 10^−2^ mW mm^−2^ in RGCs when spontaneous pathological activity is blocked. For comparison: The midday irradiance on a surface in the Sahara Desert is 10^0^ mW mm^−2^. The peak irradiance in London on a typical summer day is around 10^−1^ mW mm^−2^, and the irradiance in a well-lit room is 10^−4^ mW mm^−2^ (note the reflected irradiance reaching the retina would be around four orders of magnitude less).

**Table 1.** Summary of optogenetic retina radiance requirement.

Cell type	Irradiance range (mW mm^−2^)	S-curve midpoint (mW mm^−2^)	Reference
Ganglion cells	10^−2^–10^1^	4.3 × 10^−1^	Bi *et al* [21]
Bipolar cells	10^−2^–10^0^	4.2 × 10^−2^	Cronin *et al* [20]
Degenerate cone cells	10^−4^–10^0^	2.5 × 10^−3^	Busskamp *et al* [19]

A further constraint is that the photons in blue light (470 nm, 2.6 eV) used to stimulate ChR2 have sufficient energy to cause photochemical damage and thus photoretinitis. As such, earlier work by ourselves [24] explored the regulatory limit [25] from the perspective of the emitter, which defines that an emitter should not have an average luminance exceeding 0.1 mW mm^−2^ · Sr over any 10 000 s period. We calculate this in the next section to define the maximum luminance of a display. We derive a luminance of 90 332 cd m^−2^ based on empirical evidence. This perhaps eases constraints on the emitter, but it is still significantly beyond the 100–1000 cd m^−2^ luminance of typical LCD and OLED displays. As such, an intensifier headset will be required.

**Figure 1.**
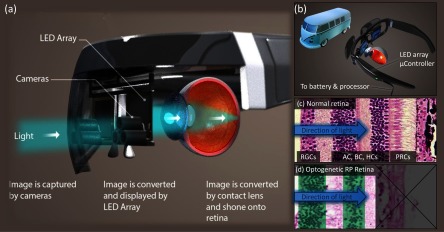
A concept diagram of how a headset could be constructed to provide optogenetic forms of retinal prosthesis. (a) and (b) A headset with cameras and processor (not shown) would acquire the visual scene and process prior to driving high radiance LED arrays. Optics (in this image a simple lens) would then transmit to the eye and through to the retina. (c) A normal retina with communication (RGC), processing (AC, BC, HC), and phototransduction (PRC) cells. (d) A diseased retina with conceptually either the retinal ganglion or bipolar cell layers photosensitized through optogenetics.

Figure 1 conceptually demonstrates how a virtual reality (VR) system can be utilised for an optogenetic version of retinal prosthesis. Cameras mounted on the headset can acquire the visual scene in either the visible, IR or UV wavelengths. Processing such as that previously described by our team [14, 26, 27] can be used initially to simplify the video signal and then to convert into a processed retinal form [26, 28, 29]. The information can then be passed to high radiance optical stimulation arrays (displays) which are then projected through VR optics to the eye. We utilise Gallium Nitride optoelectronic arrays to provide radiance at sufficient illumination as described previously in [30–32]. In this work, we demonstrate how the whole system comes together and provides sufficient radiance for optical neural stimulation.

The accommodation (i.e. near focus) of the human eye is around 12 dioptres (8 cm) for teenagers, dropping to a few dioptres (50 cm) at middle age. This means optics are required to allow focusing of miniature screens at a closer distance. Even with optics, VR headsets protrude around 12 cm from the eye compared to 3 cm for typical glasses. As such folded prism optics can bring this distance down to a more acceptable distance of around 5 cm. Clearly, for high radiance systems, the optics need to be as efficient as possible.

In the following sections. We interpret the regulatory requirements to define the display parameters for our headset. We then demonstrate a full system performance, utilising a mini-graphics processor and microcontroller system to acquire, process and transmit visual information to high-radiance gallium nitride arrays. We utilise an optic model of the eye to demonstrate that sufficient irradiance can be provided once inefficiencies in both the optoelectronics and optics are taken into account and demonstrate efficacy on a biological platform. A full table of performance versus the required criteria is defined in table 4.

## 2. Radiance limits

The large irradiance requirement of the optogenetically encoded cells coupled with losses in optical coupling from the emitter to retina would suggest that we utilise as bright a source as possible. However, there are regulatory limits defined by the EC 89/391 directive [25] (which is similar to ACGIH in the US) to prevent damage to the retina. These directives are not entirely perfect for this application. There is some critique e.g. by Vos *et al* [33] that perhaps the Netherlands Health Council guidelines dating back to 1978 are superior. Also, in more general terms damage limits to fully functional retina are perhaps not as relevant to a dysfunctional one. However, we utilise the directives as they are the current regulatory guidelines in the absence of strong contrary evidence.

In earlier work [24], we explored these limits and that the primary consideration is photoretinitis caused by to be photochemical damage due to photons in the range 400–700 nm (3.1–1.8 eV). It is, however, worth re-appraising the requirements from the perspective of the LED display as we move devices through to regulatory approval so as to define operating specifications. The specific guidance for emitters (with emittance angles greater than 11 mrads) is that over any 10 000 s period the average Luminance of an emitter should not exceed 0.1 mW mm^−2^ · Sr (100 W m^−2^ · Sr) of spectrally adjusted light within any 10 000 s period as defined by equation (1):

1 \begin{align*} \newcommand{\e}{{\rm e}} \displaystyle \left\{{{L}_{B}}\left( \lambda \right)=\int_{\lambda =400\,{\rm nm}}^{\lambda =700\,{\rm nm}}{{{L}_{\lambda }}\left( \lambda \right)\cdot {{B}_{\lambda }}(\lambda)\cdot d\lambda } \right\}\leqslant 100\,{\rm W}\,{{{\rm m}}^{-2}}\,{\rm Sr},\nonumber \end{align*}

where ${{L}_{B}}\left( \lambda \right)~$ is the effective radiance, expressed in W m^−2^ · Sr. ${{B}_{\lambda }}(\lambda)$ is the spectral weighting taking into account the photon energy dependence of the photochemical injury in the range 400–700 nm (3.1–1.8 eV). ${{L}_{\lambda }}(\lambda)$ is the spectral radiance of the source, expressed in W m^−2^ · Sr. Calculating this integral for a Blue LED with peak emission of 475 nm, we obtain a value for ${{L}_{\lambda }}$  =  0.05 mW mm^−2^ · Sr. To convert this into an average permissible LED radiance ${{R}_{{\rm LED}}}$ (mW mm^−2^) we can derive an expression from the Lambert cosine law which assumes that the profile of an LED varies with the cosine of the perpendicular emission angle. This is given in (2) below:

2 \begin{align*} \newcommand{\e}{{\rm e}} \displaystyle {{R}_{{\rm LED}}}=\pi \cdot {{L}_{B}}.\nonumber \end{align*}

Using equation (2), we find that the average spectrally corrected radiance of an LED should not exceed 0.17 mW mm^−2^. The final consideration is that we are not using a single LED, but a display of LEDs. As we project an image, some pixels will be at maximum intensity and some pixels less so. Furthermore, as we display a video in time, there will be periods when the display is on, and periods when the display is off (i.e. duty cycling). As such, the maximum radiance from a display relative to an individual pixel therein can be expressed as follows:

3 \begin{align*} \newcommand{\e}{{\rm e}} \displaystyle {{R}_{Display}}={{R}_{{\rm LED}}}/\frac{1}{{{N}_{t}}{{N}_{x}}{{N}_{y}}}\underset{t=0}{\overset{t={{N}_{t}}}{\mathop \sum }}\,\underset{x=1}{\overset{x={{N}_{x}}}{\mathop \sum }}\,\underset{y=1}{\overset{y={{N}_{y}}}{\mathop \sum }}\,\frac{{{R}_{{\rm LED}}}(x,y,t)}{{{R}_{{\rm LED{{\text -}}max}}}}.\nonumber \end{align*}

The division terms can be summarised as the average proportional pixel intensity in video display of typical scenes. We performed 3 videos of typical natural scenes using a mobile phone camera and then processed them using our simplification and retinal processing sequence described in section 5. We found that the average retinal image illuminates 12% of the maximum value, taking into account dark and bright regions. This derived figure is commonly accepted in the retinomorphic imaging community, so it is presented in the supplementary notes (stacks.iop.org/JNE/15/065002/mmedia). Furthermore, for a video rate of 25 Hz, each frame would consume 40 ms. We propose to illuminate for half that time, i.e. 20 ms as the channelrhodopsins operate more efficiently in short pulses [34]. This equates to a 50% duty cycle. As such the division term in (3) becomes 0.075, and the maximum value of ${{R}_{Display}}$ should therefore be 2.77 mW mm^−2^. If any LED is brighter, the duty cycle would need to be a proportion fraction of the 50% duty cycle. For comparison with normal displays, we can use equation (4):

4 \begin{align*} \newcommand{\e}{{\rm e}} \displaystyle {{L}_{V}}\left( \lambda \right)=683.02\frac{\eta \left( \lambda \right)\cdot {{I}_{r}}}{\pi }\frac{P}{A},\nonumber \end{align*}

where ${{L}_{V}}\left( \lambda \right)$ is the Luminance in cd m^−2^ · Sr. $ \newcommand{\e}{{\rm e}} \eta \left( \lambda \right)$ is the luminous efficacy factor, which for 470 nm  =  0.15. The *π* term in the divisor results from the steradian emission profile defined by the lambert cosine law in equation (2). *P* and *A* are the power and area correction factors to move from mW to W, and mm^2^ to m^2^, respectively. Calculating ${{L}_{V}}$ for a 475 nm emitting display from ${{R}_{Display}}$ equates to 90 332 cd m^−2^.

**Figure 2.**
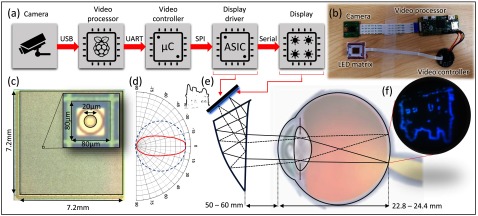
Hardware flow diagram of the proposed system (a) hardware block diagram of the proposed system and the role of each block, (b) an image of the key components. (c) An image of the 90  ×  90 matrix array with a close-up of a single LED-pixel. Each pixel area is 80  ×  80 *µ*m, and within that, the LED emitter has a diameter of 20 *µ*m. (d) The emitter profile of typical Lambertian LEDs (blue dots), and our narrow emission LED (red). (e) An optical diagram of how the matrix integrates into a VR optical system and resultant image (f).

For our optical system, we utilised had a 4 mm aperture for both the optical system and the eye pupil. Additionally, the *f*# (focal length divided by the aperture) of the optical system was 5.5, thus the emission angle is 10.4°. Given that the radiance profile of our LEDs is tighter than for normal LEDs (see figure 2(d)), the median optical coupling efficiency for our system is 5.75%. If the pupil diameter was to constrict to a minimum of 2 mm (see discussion), then it would become the effective aperture. In this scenario, then the optical efficiency would be expected to drop to 1.44%. As such the maximum irradiance on the retina derived from ${{R}_{Display}}$ will be to be 0.17 mW mm^−2^ in the case of the measurements from a 4 mm pupil, and 0.04 mW mm^−2^ in the case of a 2 mm pupil. These calculations are summarised in table 2 below.

**Table 2.** Radiance specifications derived from the regulatory directives.

Condition	Max value	unit	Notes
${{L}_{B}}\left( \lambda \right)$	0.1	mW mm^−2^ · Sr	Maximum average luminance from an emitting source in any 10 000 s period
${{L}_{\lambda }}\left( \lambda \right)$	0.05	mW mm^−2^ · Sr	Maximum spectrally adjusted average luminance from a 475 nm source (including the full emission spectrum profile for our LEDs)
${{R}_{LED}}$	0.33	mW mm^−2^	Maximum average radiance from a Lambertian emitting LED
${{R}_{Display}}$	2.77	mW mm^−2^	Maximum peak radiance for a Lambertian display with an average integral image at 12% this peak intensity and a duty cycle of 50%
${{I}_{Display}}$	0.17	mW mm^−2^	The equivalent irradiance on the retina for optical system assuming optical coupling efficiencies of 5.75% (top for 4 mm pupil) and 1.44% (bottom, 2 mm pupil)
	0.04		

## 3. System architecture

**Figure 3.**
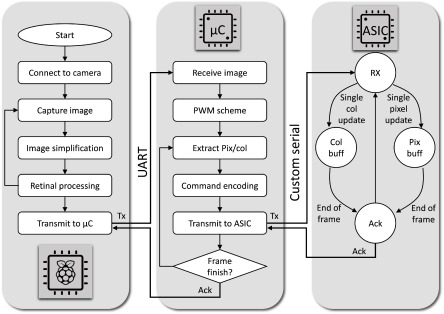
Software flow chart for each stage in the system. (Left) The flowchart for the image simplification and retinal processing software on the Raspberry Pi videoprocessor. (Centre) The flow chart of the firmware on the microcontroller (right) the state machine of the CMOS ASIC chip controlling the *µ*LED matrix.

The system is depicted in figure 2 and consists of three main parts; The first is the (opto)electronics, which includes a camera, video processor, matrix microcontroller, and high radiance LED matrix, and is illustrated in figures 2(a)–(c). The second part is the firmware, which includes image processing on the video processor and matrix control on the microcontroller and is illustrated in figure 3. The final part is the optical system which consists of the VR lens to focus the output pattern on the eye and is illustrated in figures 2(d)–(f).

## 4. (Opto)electronic hardware

The optoelectronic system flow is described in figure 2(a) with an image of the components in figure 2(b). It consists of five main parts; A camera, a videoprocessor (Raspberry Pi), a microcontroller (NXP LPC 4330), and custom Application Specific Integrated Circuit (ASIC, previously described by us in [31]) which is flip-chip bonded to a Gallium Nitride microLED array. Details of the LED can be found in [35]. The Raspberry Pi board is used in this work because of its compactness, built-in camera interface and ease of programming and compatibility with MATLAB.

The ASIC-LED matrix array described here is an evolution on earlier models [31, 35, 36], which improved bonding and efficiency. In brief, the CMOS chips have been fabricated using xFAB 0.35 *µ*m technology. The microLED arrays have been custom fabricated from Gallium Nitride LED wafers and flip-chip bonded to the CMOS ASIC. It has a serial interface which allows driving of individual LEDs or individual rows of LEDs, typically the latter. This is achieved with 90 bits per row equating to 8100 bits (~1 kilobyte) per refresh. Intensity can be globally modulated to a fixed maximum radiance through a voltage setting; then each LED can be modulated through pulse width modulation. A refresh rate of 25 Hz and a 5-bit intensity modulation can thus be achieved for a data rate of 7.2 Mbits s^−1^. Variances in emissivity between LEDs can be calibrated out within the PWM scheme.

Improving on prior efforts [31, 32], we have developed a custom board primarily consisting of an NXP LPC 4330 microcontroller, power management, and power regulators to provide stable voltage supplies to the Matrix ASIC and LED array. Power can thus be taken either from a USB power supply (5 V) on the video processor unit or more efficiently directly from the Lithium Ion battery supply (3.7 V). The microcontroller board communicates with the matrix a serial clock of 7.2 MHz. Hence, a single data burst takes 12.5 *µ*s while the whole frame (90 rows) takes 1.13 ms. So, the minimum PWM dynamic range within the target 20 ms duty cycle is 17 levels. However, with PWM, not all the pixels need to be updated each frame, so it is possible to achieve ~5 bits of dynamic range in intensity modulation. Though the caveat is that some of this needs to be used to reduce mismatch between pixels.

Passing information to the matrix microcontroller is the camera and videoprocessor. For the latter, we have utilised a Raspberry Pi v3 unit, which fundamentally consists of a Quad Core 1.2 GHz Broadcom BCM2837 64 bit CPU, and a VideoCoreIV-AG100-R GPU which is OpenGL compliant. In figure 2(b) we demonstrate this with the Raspberry Pi Zero, which has a slightly slower BCM2835 chip but is considerably more compact. The development was carried out on the V3 system but can perform on both. The interface between the video processor and the microcontroller is via a UART interface.

## 5. Software architecture

The system software comprises three primary parts as illustrated in figure 3. The first stage involves video processing which is an adapted implementation of our prior proposed scheme in [26]. This is implemented on the (Raspberry Pi) videoprocessor unit and is illustrated in figure 3 (left). The second stage involves the firmware to convert individual images into a video display driving scheme to be sent to the matrix. This has been implemented on the (NXP LPC 4330) microcontroller and is illustrated in figure 3 (centre). The final part is the state machine on the CMOS chip as shown in figure 3 (right).

It remains to be seen how optogenetic forms of retinal prosthesis compares in clinical efficacy to electronic forms. But it is unlikely that perfect high resolution will be returned in the first instance. We have therefore previously proposed schemes to simplify the presented information into more cartoon-like forms [14, 26]. The cartoonisation algorithm firstly converts to grey. Then it utilises canny edge filtering to highlight key edges of the scene and anisotropic smoothing to remove less important textures. We have also previously explored extra spectral imaging such as IR and UV to enhance the scene [37], but for simplicity, do not utilise it here.

The next stage is the simulated retinal processing. Rather than attempt to reproduce the full suite of retinal computations, we utilise a simple spatio-temporal filter based on a difference of Gaussians filtering, to mimic the centre-surround architecture of the inner retinal network. This is performed utilising a pyramidal scaling approach (described in more detail by Burt *et al* [38]) which allows the use of computationally efficient 3  ×  3 kernels with downscaling to traverse spatial frequencies. Temporal filtering is performed over a 5-frame period. These algorithms were designed in MATLAB and compiled to function in the Raspberry Pi Linux operating system. Though further improvements could be made by programming directly in C and utilising OpenGL processing, we found the performance adequate. Once complete, the information is streamed from the Pi to the microcontroller via a serial peripheral interface.

The microcontroller receives processed video frames and has to convert them into a pulse width modulated output (figure 3 centre). Furthermore, there is a variance of the emittance of individual LEDs on the microdisplay from 5 *µ*W to 100 *µ*W primarily due to the mismatch in the contact resistances of LEDs during the fabrication process as shown in figure 6(a). As such, a calibration file is used to convert individual images to a pulse width equivalent. Then, for each frame, the visual information is read out into individual rows and transmitted to the matrix via the serial interface. For particularly sparse images, data can also be transmitted on a LED-pixel basis with more common in address event representation common in neuromorphic systems. Each row/pixel command either contains a pixel ON 〈1〉 or pixel OFF 〈0〉 instruction per pixel. Transmission is over a custom serial interface which is bit-banged from the microcontroller.

The LED matrix has a state machine (figure 3 right) to receive the incoming data and determine whether it refers to updating an individual row (long shift mode) or (pixel shift mode). Information is address-based so each row or pixel can update randomly. It is thus possible for the microcontroller to send mixed information (e.g. pixel and/or full row) for each row, and skip rows where updates are not required. This can both increase the temporal resolution as well as reduce power consumption in transmission. A more detailed description of the matrix data burst structure is discussed in [31].

## 6. Optical system

The optical system needs to mechanically hold the other components and optically deliver light from a high radiance display into the eye. It needs to be compact and wearable, as such, the protrusion from the eye needs to be minimized. For comparison standard glasses typically protrude 2–3 cm and can mechanically rest largely on the bridge of the nose. We explored two possibilities—a linear VR lens from the Oculus Rift VR system and a folded WFO-5 prism lens from an eMagin z800 VR headset. Of these we found the eMagin prism lens to be the best in terms of performance and compactness—with placement 6 mm from the eye.

The prism VR lens has an optical profile described in detail by Cheng *et al* [39] and is illustrated in figure 2(e). In brief, it has to two optical paths. The first optical path is formed by a wedge-shaped freeform surface (FFS) prism which is built from three physical surfaces as illustrated in figure 2(a). The top surface is at an angle from the display to receive the light. The front surface is a half-mirror to allow the rays to be reflected within the prism. The rear surface acts as an even ‘asphere’. Exemplar ray traces demonstrate the optical path through the system. In normal multicolour VR systems, such optics have notable chromatic distortions at the periphery, which need be calibrated out in the display software. But that is not applicable in this case as the source is monochromatic. The output of the prism optic is at infinity. As such, it may be that individuals with particularly short vision may need to utilise the system in tandem with contact lenses.

Optically, the prism VR lens has a field of view of just under 40°. Though as our microdisplay is smaller than the originally designed OLED display, the FoV is somewhat smaller (not measured). More importantly, the *f*# of the VR prism lens is 5.5 with an effective focal length of 22 mm. Thus, the exit pupil (effective focal length/*f*#) is 4 mm, which matches the constricted eye pupil as described in the experimental setup section below.

The LED microdisplay display dimensions can be seen in figure 2(c). We adapted a PCB mount to replace the original OLED display that eMagin utilised with the prism optics. A further notable feature is that the micro-LEDs have been designed with a narrow emission profile compared to standard Lambertian emission for LEDs as can be seen in figure 2(d). This means the optical efficiency is higher for such a system.

## 7. Experimental setup

**Figure 4.**
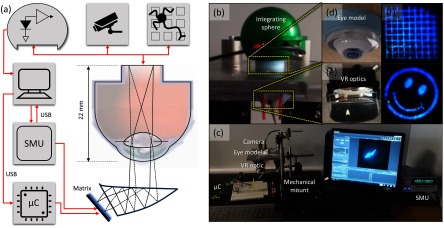
A diagram of the test system (a) simplified diagram of the test platform of the matrix, showing the connections between different testing equipment and the *µ*LED matrix, VR lens and optical model of the eye. (b) and (c) Photo of the platform used for the matrix, eye model and the mechanical system, (d) photo of the eye model which has been adapted to provide a reverse aperture. (e) Mechanical mount for VR prism optics. (f) Exemplar output images after the eye and on the top of the image sensor show a very good resolution for the received image.

The key advance in this paper is to demonstrate that the fully developed optical system can deliver sufficient light into an optogenetically modified retina. We, therefore, developed an experimental test platform that is illustrated in figure 4 below. We purchased an optical model of the human eye (OEMI-7, Ocular instruments) which has the same dimensions and optical properties of the eye including a water-filled vitreous humour. The dimensions of the model accurately mimic the eye. However, the pupil size is 7 mm, which equates to a dilated eye. We modified the model by 3D printing a new rear piece with an aperture which can be attached to a sensing mechanism. An image of this is shown in figure 4(d). The light blue rear section can be rotated modify the internal distance between lens and aperture to compensate for sensor thickness.

It should be noted that the eye model we utilized is designed for optometry training and thus has a dilated pupil, thus allowing in the maximum amount of light. However, as the exit pupil of the VR lens was 4 mm, This becomes the primary limiting aperture of the optical system. However, there is some question as to whether in operation with humans the pupil could constrict further. Birren *et al* [41] have shown that the pupil size varies from [5.1, 7.4] mm in [light, dark] for individuals in their twenties to [3.4, 4.9] mm for individuals in their eighties. However, it can be argued that for the extreme bright intensities that are being emitted from our device that the pupil could constrict to 2 mm [44–46]. The counter argument is that even though pupil size is partially controlled by intrinsically photosensitive RGCs [40], there is a body of evidence in the literature that the amplitude of the response are both significantly reduced in photoreceptor dystrophies [47–49]. As such, we have measured results for the effective 4 mm pupil size of our experimental system, and then calculated what the performance would be for a 2 mm pupil size in the extreme case.

We used three different sensing mechanisms: (i) An integrating sphere (OMP150, Artifex engineering) to measure exact irradiance through the aperture (ii) a scientific camera (xiQ MQ042CG-CM, Ximea) chip, i.e. optics removed, to measure irradiant distribution (i.e. image). (iii) A microelectrode array (Multichannel systems MEA64) with an optogenetic rodent retina to demonstrate sufficient sensitivity on retina.

We used a source measure unit (2612B, Keithley Instruments) to monitor the current through individual LEDs to determine efficiency. The full arrangement of this is described in the cartoon figure 4(a). The mechanical mounting arrangement can be seen in figures 4(b) and (c). The VR optical prism lens and mechanical mount can be seen in (e). Sample images can be seen in (f). In the latter, some barrel distortion can be seen in the grid image. This is expected as the optics of the eye are designed to focus on a curved surface, whereas our aperture is flat.

Biological experiments were carried out with whole retina extracted from Rd1 mice cross-bred with Thy1-ChR2 expressing mice to provide model of retinal degeneration with optogenetic neural control. The protocol was the same as that used in [42]. Mice were killed by cervical dislocation and their eyes quickly enucleated and placed into room temperature artificial cerebrospinal fluid (aCSF) containing (in mM) 118 NaCl, 25 NaHCO_3_, 1 NaH_2_PO_4_, 3 KCl, 1 MgCl_2_, 2 CaCl_2_, and 10 glucose, equilibrated with 95% O_2_ and 5% CO_2_ for retinal dissection. The isolated retina was placed wholemount, RGC layer facing down, onto a 60-channel indium tin oxide multielectrode array (MEA; Multichannel Systems, Reutlingen, Germany). A small piece of polyester membrane filter (5 mm pores) (Sterlitech) and a diamond- or ring-shaped metal weight (Warner Instruments, Hamden, CT, USA) were placed on the retina to improve coupling between the tissue and the electrodes. Once in the MEA chamber, the retina was kept at 32 °C and continuously perfused with aCSF at 1–2 ml min^−1^. The retina was allowed to settle for 2 h before any recordings were taken. Electrophysiological activity was recorded at a sampling rate of 25 kHz using MC Rack software (MultiChannelSystems). Spikes were extracted from high-pass filtered data (cut-off 300 Hz) by applying a voltage threshold, independently set for each channel to seven standard deviations below a 60 s baseline recording from the empty MEA and adjusted manually to maximise spike detection while minimizing noise. Spike waveforms comprising 16 samples before and 32 samples after each threshold crossing were extracted and imported into Offline Sorter (Plexon, Dallas, USA) for spike sorting. Automatic spike sorting was performed using T-distribution Expectation-Maximization [43], followed by manual inspection to ensure accuracy of sorting. To ensure that we were only receiving responses from the RGCs, we applied 20 *µ*M DNQX and 10 *µ*M D-AP4 to block synaptic inputs from other cells.

These experiments were carried out prior to the later experiments and used a lower density 16  ×  16 LED array rather than the 90  ×  90 LED array. The lower density array was built on similar technology to the 90  ×  90 array but differed in three ways (i) Using an older technology the LEDs were not as efficient (ii) the LED spacing was 150 *µ*m, and thus the stimulus pixel area was 22 500 *µ*m^2^, i.e. for the same LED emission, the irradiance was 3.5  ×  more diffuse. (iii) To partially compensate the lower density array, we utilized a high voltage technology allowing much higher drive voltages. Given points (i)–(iii) the lower density array could provide a median peak irradiance on the retina of 0.1 mW mm^−2^ compared to a median peak irradiance of 1.35 mW mm^−2^ for the high density 90  ×  90 array. However, given the regulatory limits defined in section 2, we wanted to explore to what extent it was possible to stimulate optogenetic retinal cells in the lower range of 0.01–0.1 mW mm^−2^.

## 8. Results

**Figure 5.**
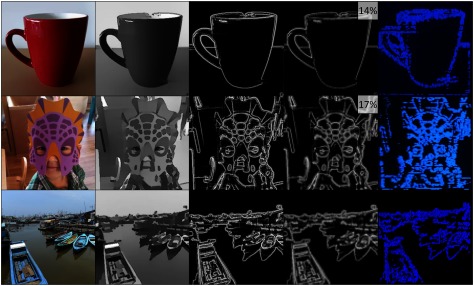
Image processing flow of increasing complexity from the top (cup) through to bottom (boat). From left to right, are original image (at 270  ×  270 pixel resolution), simplified, contrast-enhanced image (at 270  ×  270 pixels resolution), retinally processed image (at 270  ×  270 pixel resolution), retinally processed image (at 90  ×  90 pixel resolution). Finally, the retinally processed image is displayed in the LED matrix.

Results from the image processing flow can be seen in figure 5. These illustrate the key steps in our processing functions, and perhaps more importantly to this paper, how the processed data would look on the optoelectronic display. Three images are presented with increasing complexity from top to bottom, i.e. the minion image is already a cartoon and thus has very limited texture. The mask image and boat scene images were both taken utilising mobile cameras, with the latter much more complex due to background vegetation and multiple faces. The left-most column in figure 5 presents the original image downscaled to a resolution of 270  ×  270 pixels (i.e. 4×  the display resolution). The second column presents the image simplification effect. The third column presents the spatial aspect of the simulated retinal processing. The fourth column shows the retinal processing downscaled to the native 90  ×  90 resolution of the high radiance display. The final column shows the same image from the display itself. We were able to achieve a full processing stream from camera to display at 25 Hz, with a total current consumption averaging at 377 mA [Camera: 52, videoprocessor 150, video controller: 31, power management: 4, 2×  LED matrix: 140 mA]. Using a typical 10 000 mAhr Li-Ion battery found in typical tablets, (weight: ~200 g), this would allow  >24 h of use between recharging.

**Figure 6.**
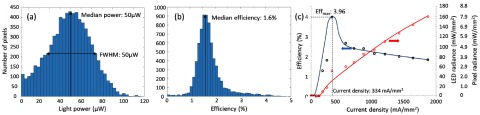
Summary of the measured data for the optical matrix emitter (a) measured output light per LED and the median output light is 50 *µ*W at 5 V LED driving voltage, (b) efficiency per LED and the median LED efficiency is 1.6% at 5 V LED driving voltage, (c) measured mean radiance and efficiency with varying current density. Radiance is provided for both the LED and the larger emitting pixel. The average peak efficiency is 3.96% at a current intensity of 334.4 mA mm^−2^ and voltage of 3.8 V.

The optoelectronic performance of the matrix prior to use in the optical system can be seen in figure 6(a), which shows the optical radiance from each LED. The maximum power per LED is around 100 *µ*W and median power at 50 *µ*W with a full width half maximum also at 50 *µ*W. Each LED has a diameter of 20 *µ*m (area  =  314 *µ*m^2^). Thus, the median radiance per LED is 160 mW mm^−2^. For reference, it is useful to compare with standard display technology using equation (4) this would give a peak luminance of 5  ×  10^6^ cd m^−2^. However, considering purely the LED luminance in practice provides an artificially large value. i.e. the fill factor of the stimulus-pixel also needs to be considered.

The LED diameter is 20 *µ*m (area 314 *µ*m^2^) compared to our pixel area of 80  ×  80 *µ*m (6400 *µ*m^2^). The median radiance per stimulus-pixel is therefore 7.8 mW mm^−2^ (254 365 cd m^−2^). Both LED and pixel radiance values are provided on the right-*y* axis in figure 6(c). This is higher than the regulatory limit and represents the maximum demonstrable value. In practice, we would therefore either limit the peak radiances or peak duty cycle for each of the LEDs. It is also worth noting that stimulus-pixel can be effectively filled through out-of-focus blurring. Also, on the retina, the optics de-magnifies the stimulus-pixel area to 2268 *µ*m^2^ (48  ×  48 *µ*m).

Figure 6(b) presents the wall-plug efficiency of the array including drive electronics. The median wall-plug efficiency is 1.5%, which is an improvement upon our earlier work which presented  <1% [29, 30]. Figure 6(c) presents the measured emittance and efficiency of an exemplar LED as a function of current density. The average peak efficiency is 3.96% at a current intensity of 334 mA mm^−2^ and voltage of 3.8 V. The primary reason for the variation in the emittance shown in (a) and (b) is due to sheet/contact resistances in the LED bonding process which determine variations in voltage requirement.

**Figure 7.**
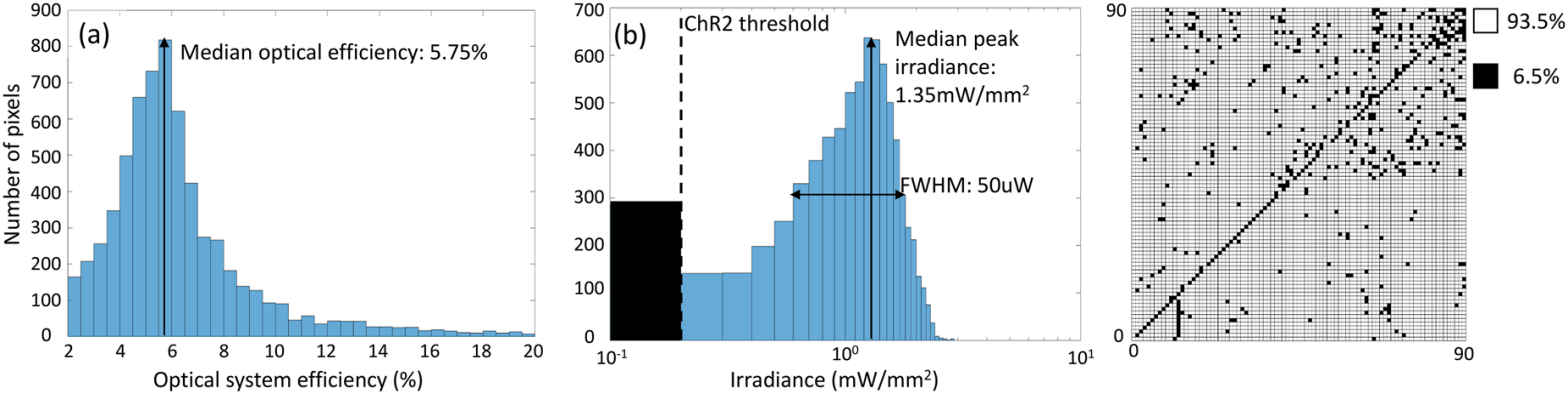
The performance of the optical system (a) a histogram of the efficiency of the optical system per pixel. The mean optical efficiency is 5.75%. (b) The measured irradiance density per pixel after the optical system and the eye model, using a measured pixel size of 2268 *µ*m^2^ on the retina. The median peak irradiance density from the display on the retina is 1.35 mW mm^−2^. (c) An image distribution of the irradiance split into two forms: white representing pixels of sufficient irradiance density (i.e.  >0.1 mW mm^−2^ for our experimental 4 mm pupil size), and black pixels representing those that are not expected to stimulate optogenetic retinae (i.e  <0.01 mW mm^−2^).

Figure 7 shows the results of the optical system experiments to determine the optical characteristics in the retina. Figure 7(a) shows a histogram of the measured optical efficiency. These were calculated by dividing the recorded irradiance (measured by the integrating sphere) by the output radiance from each LED. The median optical efficiency is 5.75%, with peak efficiencies approaching 20%. There was no specific pattern in the variance, and we believe it to follow a standard measurement error distribution around the mean recorded value. Given the *f*# of optical system, and thus an emission angle of 10.4° versus the emission profile presented in figure 2(d) we believe this peak efficiency is probably close to the maximum for our system. The efficiency spread thus represents experimental error.

Figure 7(b) shows the resultant irradiance density histogram of each optical stimulus-pixel on the retina. We used the camera to determine the area of each stimulus-pixel. This was determined by integrating the camera-pixels and multiplying by the camera-pixel size (75 pixels  ×  5.5 *µ*m  ×  5.5 *µ*m  =  2268 *µ*m^2^). Exemplars of the image on the retina can be seen in figure 4(f). One caveat to this approach is that the optics of the human eye and our eye model are designed to focus on a curved retinal surface. However, in our setup, the camera surface is flat. Hence there was some noticeable barrel distortion that can be seen in the grid pattern in figure 4(f).

The irradiance density of each optical stimulus-pixel was then calculated by dividing the measured retinal irradiance by the pixel size. The median peak irradiance from the display on the model retina is 1.35 mW mm^−2^. This is ~2×  higher than the accepted ‘threshold’ for stimulating dissociated cells: 0.7 mW mm^−2^ [22]. However, our experimental system—have matched exit and entrance pupils (i.e. for VR optics and eye) of 4 mm. It could be argued that for extreme light intensities, the eye pupil can constrict to 2 mm. In this scenario, the median peak irradiance on the retina would be expected to drop to 0.34 mW mm^−2^. This is lower than the accepted ‘threshold’, but certainly well within the range of stimulation demonstrate in table 1.

The variability in output is primarily the result of imperfect bonding between the LED array and the CMOS chip, leading to variabilities in the contact resistances which reduce efficiency. This can be corrected through modification of the pulse width modulation. i.e. driving the brighter pixels for shorter and the dimmer pixels for longer within any given video frame and target PWM intensity.

Although photosensitized RGCs have photoresponses in the range 10^−2^–10^1^ mW mm^−2^, we need to consider a wide operational dynamic range to bring back effective vision. We, therefore, define two domains: ‘*viable*’ (white, >0.1 mW mm^−2^) and ‘nonviable’ (black, <0.1 mW mm^2^) within our experimental system with a 4 mm pupil size. For our array, we found 93.5% were capable of reaching or exceeding this defined range. We would expect 100% could be achieved in a commercial manufacturing process. The pattern of variability can be seen in figure 7(c). We believe the diagonal line to be a bug in the CMOS state machine.

**Figure 8.**
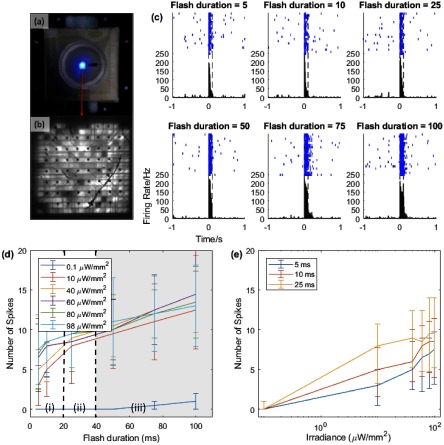
Experimental results on biological retina. (a) and (b) Images of a 16  ×  16 array on the retina mounted on an MEA plate. The optic distribution was slightly different from the experiments with the 90  ×  90 array, but the average irradiance density was similar at 1.43 mW mm^−2^. (c) Raster plot and peri-stimulus time histogram of an example RGC response to 4 V LED flashes. Each blue line represents one spike event (each row shows one trial), and the histogram shows the average firing rate in each 10 ms bin. The stimulus starts at 0 s, and the black dashed lines show the 100 ms window over which the response is calculated. (d) Median response of all responsive cells to LED flashes of different durations and intensities. Error bars are an interquartile range over cells. The three regions: (i) represents the primary stimulus period, (ii) is the maximum period for 25 Hz video, (iii) would not fulfil 25 Hz video but is shown for completeness. (e) Data from (d) for flash durations of 5–25 ms plotted as a function of irradiance.

The final experiment was to demonstrate that the present calculations are indeed sufficient to stimulate an optogenetic retina. The isolated mouse retina was placed on an 8  ×  8 microelectrode array matrix, so we simply presented full field illumination at different pulse widths and voltages and measured the resulting RGC firing rate at different electrodes on the micro-electrode array. Each pulse width was presented 20 times in randomised blocks with a 2 s inter-stimulus interval (ISI) at a fixed voltage, then repeated for the next voltage. An image of the illumination can be seen in figures 8(a) and (b), along with raster plots of responses from an example RGC (c). Note this was done with the earlier 16  ×  16 matrix and with a lower irradiance operational range on retina of 0.01–0.1 mW mm^−2^.

To quantify the responses, we bootstrapped a null distribution of responses by placing fake stimulus timestamps with the same ISI statistics starting at random points within the data and asked how many cells ‘responded’. Here, a response was defined as firing at least one spike per trial and increasing firing rate during the 100 ms after the stimulus compared to the 1 s before the stimulus. This was repeated 6000 times and the fraction of ‘responsive’ cells calculated. Next, we calculated the fraction of RGCs that actually responded to the odd-numbered 100 ms flashes at each voltage. Finally, we calculated the probability of observing that many responsive cells by chance using the null distribution. The results, shown in table 3, confirm that RGCs significantly responded to LED illumination.

**Table 3.** RGC response statistics. Middle column shows fraction of responding RGCs (out of a total of 53 isolated single units) as a function of LED voltage, given in the left column. Right column gives the *p*-value calculated from the bootstrap test (see text).

Voltage setting (V)	Irradiance (mW mm^−2^)	#Responding RGCs	*P*
3	0.0001	8/53	0.0032
4	0.01	42/53	<0.0002
5	0.04	42/53	<0.0002
6	0.06	44/53	<0.0002
7	0.08	44/53	<0.0002
8	0.1	44/53	<0.0002

To assess the relationship between LED voltage, flash duration, and RGC spiking, we analysed the responses to the even-numbered flashes of all flash durations, which are shown in figures 8(d) and (e). The number of spikes per flash increased significantly with both voltage (Friedman test on grand median across trials and cells, pulse width as blocking factor; *χ*^2^ (5)  =  22.52, *p*  =  0.0004) and pulse width (Friedman test, voltage as blocking factor; *χ*^2^ (5)  =  28.75, *p*  =  0.000 03). The largest increase in RGC firing with voltage appears to happen between 3 and 4 volts and, above this voltage, strong responses can be seen at all pulse widths tested. Note that for a 25 Hz video refresh, the stimulus would need to be within 40 ms.

## 9. Discussion

A simple summary of this work is that we can demonstrate that compact high radiance gallium nitride displays can be used with conventional VR optics to stimulate an optogenetic retina. We have used an optical system with prism VR optics matched to a pupil diameter of 4 mm, which is what is available in our model eye. It is difficult to physically modify the aperture but for small angles, we expect the light throughout to scale with area. It can be argued that for the extreme bright intensities that are being emitted from our device that the pupil could constrict to 2 mm [44–46]. In such a scenario, the light intensity would reduce by 4 times. It is however worth remembering that pupil constriction is normally controlled by rods, cones and melanopsin RGCs. Therefore, the threshold to elicit pupillary constriction and the amplitude of the response are both significantly reduced in photoreceptor dystrophies [47–49]. Since optogenetic stimulation of retinal neurons would be implemented only in patients with advanced photoreceptor dystrophies, the reduction in light intensity (necessary to activate ChR2-expressing cells) due to pupil constriction would be much less pronounced in these patients than in a healthy eye.

We show using an optic model of the eye that we can achieve a median retinal irradiance of 1.35 mW mm^−2^, which equates to 0.34 mW mm^−2^ if a 2 mm pupil is used. However, we show that such irradiances are much higher than what is suggested by the regulatory literature. The regulations provide guidelines from the perspective of the emitter, and do not consider pupil size. They could also be critiqued as not being able to refer to any long-term study on the effect of blue light on photochemical retinal damage. Furthermore, there is an open question as to how relevant photochemical damage profiles on a healthy retina are to a degenerate one. However, in the absence of contrary evidence, the existing rules would need to be used with a conservative pupil size of 2 mm. As such, for our optical system, the recommendations would be for a peak irradiance of 0.04 mW mm^−2^. This is significantly lower than the normally expected ‘threshold’ for channelrhodopsin encoded cells. We therefore We show both in terms of the literature (table 2) and our own results shown in figure 8 than intensities below 0.1 mW mm^−2^ are sufficient to determine retinal activity. Our rationale is that the typically quoted ‘threshold’ is a measure to attain 50% of the maximum firing activity in dissociated culture. For tissue, where the connectivity is still intact, thresholds are much lower, as demonstrated by table 2.

The long-term direction for this field is therefore to perform two things:

(1) Increase the sensitivity of opsins: There have been demonstrations of opsins with significantly lower light thresholds than wild-type channelrhodopsin—e.g. CatCh [22]. Additionally, if bipolar cells can be utilized rather than RGCs, the operational range drops by an order of magnitude [20]. It may also be the case that if support cells such as astrocytes are photosensitized their feedback to retinal neurons may reduce the activation irradiance on those cells [50]. The effect of more sensitive opsins would be to increase the dynamic range of neural activity and thus image contrast within regulatory limits.
(2) Red shift the opsins: The regulatory limits have a strong wavelength dependency. So, for example, shifting the opsin sensitivity and thus illumination wavelength peak from 475 nm to 550 nm would allow 55×  more light into the retina. Shifting it further to 570 nm would increase the threshold by 174×  . The caveat is that optical technologies would need to be adapted to match such developments.

Finally, it is worth considering the long-term direction and possibilities for the field. We have utilized the regulatory protocols for a non-narrow beam emitter. But some of the other rules from the perspective of the retina, particularly for coherent sources may be tougher. As such, there needs to be some long term studies to explore damage as well as reduce the effect as highlighted above. But there is also an ergonomic issue that in individuals with some remaining photoreceptors, the intense blue light may cause discomfort—at least until the remaining photoreceptors are bleached. Then there is the further issue of intrinsically photosensitive RGCs which modulate the awake-sleep cycle. Will they be affected by overstimulation? Again, this is something to be explored in the ongoing clinical trials.

We do not believe the imaging array needs to become significantly more radiant given regulatory limits. But efficiency could improve. The peak optoelectronic wall-plug efficiency of the LEDs could in future be improved from its current median of 1.5%. Best in class Gallium Nitride LEDs can approach peak efficiencies of ~80%. For example, Narukawa *et al* [51] demonstrated peak wall plug efficiencies of 85%, albeit with white light emission and current densities of 150 mW mm^−2^ on a mini 450  ×  450 *µ*m LED. More moderate efficiencies of 35% were demonstrated by Narukawa *et al* [52] and Schmidt *et al* [53], respectively, with current densities of 20 000, and 1111 mA mm^−2^, which is in the range of what we present here. Similarly, typical lighting LEDs now approach 40% efficiency. Furthermore, improvements in the manufacturing process could certainly improve the efficiency of the bonding process. In particular, the CMOS control chip was designed to operate at 5 V however currently the bonded LEDs require around 4–4.5 V to operate compared to 3–3.5 V for an LED only at the required radiances. This means that the drive transistor is operating in triode mode rather than saturation (ideal current driver). Thus, with some moderate improvements in LED and bonding efficiency, the LEDs could be fully current driven, which would result in a much higher level of uniformity.

Another aspect to consider is the use of better optics and micro-optics to improve the optical efficiency. We utilised *µ*LEDs with back reflectors more fully described in [35], with an emissive arc shown in figure 2(d). Although this is an improvement typical Lambertian emission for LEDs, a tighter arc could further improve efficiency. This could be achieved in the future with the use of surface microlenses, which could also help make the irradiance profile on the retina more uniform. However, it should be noted that improving the optical efficiency through increased collimation, would allow for improved throughput, but could fall foul of much stricter guidelines for collimated (laser) sources. Again, as noted above, there needs to be a concerted effort in the biological community to determine the long term effective of photochemical damage on degenerate optogenetic retina.

Another point to consider is the long-term improvement in both microprocessor and microcontroller technology. We have utilized the Raspberry Pi due to its elegant and compact architecture. Similarly, the videocontroller we have developed is off-the-shelf. Continued developments in this field will allow the addition of machine learning routines to further improve the cartoonisation techniques to present the most salient and useful information to the reader. Furthermore, work needs to be done on the pulse width modulation to improve the dynamic range of presented optical intensities after mismatch is taken into account. Finally, we have added a relatively simple retinal encoder in our system. Rather than trying to reproduce the full suite of retinal computations, we have put more emphasis on image simplification. The justification for this is that more advanced retinal processing becomes increasingly computationally expensive with high effective resolutions. Further, different aspects of retinal processing are subserved by distinct RGC types and circuits [54, 55], and trying to exactly simulate retinal encoding without knowing the precise cellular identity and location of the RGCs being stimulated may make the visual information conveyed to the brain more confusing, not less. Though our display is certainly higher resolution than current electronic prosthetic devices, the effective resolution remains to be seen. More advanced schemes, however, have been proposed by e.g. Nirenberg and Pandarinath [29].

## 10. Conclusion

In this work, a headset for restoring vision to those with degenerative retinal disorders has been presented. We demonstrated the system ability by building a test platform based on an eye model to measure the amount of the light on the retina surface. We demonstrate a median peak irradiance sufficient to stimulate ganglion cells on the retina. Moreover, the system is able to deliver images to the retina with a 25 frame s^−1^ including the different steps of image processing. We also demonstrate that it can be used effectively in a dimmer range limited by the regulatory guidance and still achieve effective stimulus of neural cells. Nevertheless, we do highlight that the field needs to move to redder wavelengths and continuously improve the fundamental sensitivity of the opsins to ensure photoretinitis does not occur. The headset provides a full processing flow from the camera to an optical presentation on the retina. A demonstration has been provided in an opto-biological model experiment in short-term experiments. We, therefore, hope that this technology can be utilised in upcoming human trials of optogenetic retinal prosthesis. Table 4 summarises the capability of the system versus required specifications.

**Table 4.** Summary of the proposed system performance.

Parameter	Target value	Value	Units
System performance			

Pixel resolution	>2024	8100 (93.5% functional)	Pixels
Pixel luminance	<90 332 (average)	254 365 (peak)	cd m^−2^
Median max retinal irradiance	^a^10^−3^–10^1^	1.35/0.34 (4 mm/2 mm pupil)	mW mm^−2^
PWM dynamic range	>3	4–5	Bits
Video refresh rate	⩾25	25	FPS
Total power consumption	<2000	377 (1885 @ 5 V)	mA (mW)
System weight including battery	<500	~450 (250 on head)	g

Additional parameters			

Optical efficiency	High	Median 5.75%	
LED array efficiency	High	Median 1.5%, peak 4%	

a But must also fulfil the maximum criteria set by the regulatory requirements.

## References

[1] Bourne R R A (2017). Magnitude, temporal trends, and projections of the global prevalence of blindness and distance and near vision impairment: a systematic review and meta-analysis. Lancet Glob. Health.

[2] Thylefors B, Negrel A D, Pararajasegaram R, Dadzie K Y (1995). Global data on blindness. Bull. World Health Organ..

[3] Weiland J D, Humayun M S (2014). Retinal prosthesis. IEEE Trans. Biomed. Eng..

[4] da Cruz L (2016). Five-year safety and performance results from the Argus II Retinal Prosthesis System clinical trial. Ophthalmology.

[5] Edwards T L (2019). Assessment of the electronic retinal implant Alpha AMS in restoring vision to blind patients with end-stage retinitis pigmentosa. Ophthalmology.

[6] Shivdasani M N (2017). Identification of characters and localization of images using direct multiple-electrode stimulation with a suprachoroidal retinal prosthesis. Invest. Ophthalmol. Vis. Sci..

[7] Goetz G A, Palanker D V (2016). Electronic approaches to restoration of sight. Rep. Prog. Phys..

[8] Zhao Y, Lu Y Y, Zhou C Q, Chen Y, Ren Q S, Chai X Y (2011). Chinese character recognition using simulated phosphene maps. Invest. Ophthalmol. Vis. Sci..

[9] Cha K H, Horch K, Normann R A (1992). Simulation of a phosphene-based visual-field—visual-acuity in a pixelized vision system. Ann. Biomed. Eng..

[10] Cha K, Horch K W, Normann R A, Boman D K (1992). Reading speed with a pixelized vision system. J. Opt. Soc. Am. A.

[11] Cha K, Horch K W, Normann R A (1992). Mobility performance with a pixelized vision system. Vis. Res..

[12] Thompson R W, Barnett G D, Humayun M S, Dagnelie G (2003). Facial recognition using simulated prosthetic pixelized vision. Invest. Ophthalmol. Vis. Sci..

[13] Hu J, Xia P, Gu C C, Qi J, Li S, Peng Y H (2014). Recognition of similar objects using simulated prosthetic vision. Artif. Organs.

[14] Al-Atabany W I, Memon M A, Downes S M, Degenaar P A (2010). Designing and testing scene enhancement algorithms for patients with retina degenerative disorders. Biomed. Eng. Online.

[15] Nagel G (2003). Channelrhodopsin-2, a directly light-gated cation-selective membrane channel. Proc. Natl Acad. Sci..

[16] Bergs A (2018). Rhodopsin optogenetic toolbox v2.0 for light-sensitive excitation and inhibition in Caenorhabditis elegans. PLoS One.

[17] Klapper S D, Swiersy A, Bamberg E, Busskamp V (2016). Biophysical properties of optogenetic tools and their application for vision restoration approaches. Frontiers Syst. Neurosci..

[18] Barrett J M, Berlinguer-Palmini R, Degenaar P (2014). Optogenetic approaches to retinal prosthesis. Vis. Neurosci..

[19] Busskamp V (2010). Genetic reactivation of cone photoreceptors restores visual responses in retinitis pigmentosa. Science.

[20] Cronin T (2014). Efficient transduction and optogenetic stimulation of retinal bipolar cells by a synthetic adeno-associated virus capsid and promoter. Embo Mol. Med..

[21] Bi A (2006). Ectopic expression of a microbial-type rhodopsin restores visual responses in mice with photoreceptor degeneration. Neuron.

[22] Kleinlogel S (2011). Ultra light-sensitive and fast neuronal activation with the Ca^2+^-permeable channelrhodopsin CatCh. Nat. Neurosci..

[23] Barrett J M, Hilgen G, Sernagor E (2016). Dampening spontaneous activity improves the light sensitivity and spatial acuity of optogenetic retinal prosthetic responses. Sci. Rep..

[24] Degenaar P (2009). Optobionic vision—a new genetically enhanced light on retinal prosthesis. J. Neural Eng..

[25] European Union Directive 2006/25/EC of the European Parliament and of the Council of 5 April 2006 on the minimum health and safety requirements regarding the exposure of workers to risks arising from physical agents (artificial optical radiation) (19th individual Directive within the meaning of Article 16(1) of Directive 89/391/EEC)

[26] Al-Atabany W, McGovern B, Mehran K, Berlinguer-Palmini R, Degenaar P (2013). A processing platform for optoelectronic/optogenetic retinal prosthesis. IEEE Trans. Biomed. Eng..

[27] Al-Atabany W I, Tong T, Degenaar P A (2010). Improved content aware scene retargeting for retinitis pigmentosa patients. Biomed. Eng. Online.

[28] Nikolic K (2007). A non-invasive retinal prosthesis testing the concept.

[29] Nirenberg S, Pandarinath C (2012). Retinal prosthetic strategy with the capacity to restore normal vision. Proc. Natl Acad. Sci. USA.

[30] McGovern B (2010). A new individually addressable micro-LED array for photogenetic neural stimulation. IEEE Trans. Biomed. Circuits Syst..

[31] Soltan A (2017). High density, high radiance micro-LED matrix for optogenetic retinal prostheses and planar neural stimulation. IEEE Trans. Biomed. Circuits Syst..

[32] Soltan A, Zhao H, Chaudet L, Neil M, Maaskant P, Degenaar P (2014). An 8100 pixel optoelectronic array for optogenetic retinal prosthesis.

[33] Vos J J, Van Norren D (2005). Retinal damage by optical radiation. An alternative to current, ACGIH-inspired guidelines. Clin. Exp. Optom..

[34] Nikolic K, Grossman N, Grubb M S, Burrone J, Toumazou C, Degenaar P (2009). Photocycles of channelrhodopsin-2. Photochem. Photobiol..

[35] Maaskant P P (2013). High-speed substrate-emitting micro-light-emitting diodes for applications requiring high radiance. Appl. Phys. Express.

[36] Akhter M (2015). A LED micro-display with 90  ×  90 pixels on a 80 micrometre pitch.

[37] Al-atabany W, Al Yaman M, Degenaar P (2018). Extra-spectral imaging for improving the perceived information presented in retinal prosthesis. J. Healthcare Eng..

[38] Burt P J, Adelson E H (1983). The laplacian pyramid as a compact image code. IEEE Trans. Commun..

[39] Cheng D W, Wang Y T, Hua H, Talha M M (2009). Design of an optical see-through head-mounted display with a low *f*-number and large field of view using a freeform prism. Appl. Opt..

[40] Keenan W T, Rupp A C, Ross R A, Somasundaram P, Hiriyanna S, Wu Z, Badea T C, Robinson P R, Lowell B B, Hattar S S (2016). A visual circuit uses complementary mechanisms to support transient and sustained pupil constriction. Elife.

[41] Birren J E, Casperson R C, Botwinick J (1950). Age changes in pupil size. J. Gerontol..

[42] Barrett J M, Degenaar P, Sernagor E (2015). Blockade of pathological retinal ganglion cell hyperactivity improves optogenetically evoked light responses in rd1 mice. Frontiers Cell. Neurosci..

[43] Shoham S, Fellows M R, Normann R A (2003). Robust, automatic spike sorting using mixtures of multivariate *t*-distributions. J. Neurosci. Methods.

[44] Walker H K, Hall W D, Hurst J W (1990). The pupils. Clinical Methods.

[45] Witting M D, Goyal D (2003). Normal pupillary size in fluorescent and bright light. Ann. Emergency Med..

[46] Watson A B, Yellott J I (2012). A unified formula for light-adapted pupil size. J. Vis..

[47] Aleman T S (2004). Impairment of the transient pupillary light reflex in *Rpe65*^−/-^ mice and humans with leber congenital amaurosis. Invest. Ophthalmol. Vis. Sci..

[48] Kardon R, Anderson S C, Damarjian T G, Grace E M, Stone E, Kawasaki A (2011). Chromatic pupillometry in patients with retinitis pigmentosa. Ophthalmology.

[49] Kawasaki A, Crippa S V, Kardon R, Leon L, Hamel C (2012). Characterization of pupil responses to blue and red light stimuli in autosomal dominant retinitis pigmentosa due to *NR2E3* mutation. Invest. Ophthalmol. Vis. Sci..

[50] Berlinguer-Palmini R (2014). Arrays of MicroLEDs and astrocytes: biological amplifiers to optogenetically modulate neuronal networks reducing light requirement. PLoS One.

[51] Narukawa Y, Ichikawa M, Sanga D, Sano M, Mukai T (2010). White light emitting diodes with super-high luminous efficacy. J. Phys. D: Appl. Phys..

[52] Narukawa Y, Narita J, Sakamoto T, Deguchi K, Yamada T, Mukai T (2006). Ultra-high efficiency white light emitting diodes. Japan. J. Appl. Phys..

[53] Schmidt M C (2007). High power and high external efficiency m -plane InGaN light emitting diodes. Jpn. J. Appl. Phys..

[54] Azeredo da Silveira R, Roska B (2011). Cell types, circuits, computation. Curr. Opin. Neurobiol..

[55] Gollisch T, Meister M (2010). Eye smarter than scientists believed: neural computations in circuits of the retina. Neuron.

